# Supplementary material for: MetaRibo-Seq measures translation in microbiomes
Source: Nat Commun. 2020 Jun 29;11:3268. doi: 10.1038/s41467-020-17081-z (PMC7324362; doi:10.1038/s41467-020-17081-z)
Supplement: Supplementary file 10 — Supplementary Data 7 [file 41467_2020_17081_MOESM10_ESM.zip › File2/Confidence_VeryHigh_Taxonomy/113254_out.krona.html]

Javascript must be enabled to view this page.

members
magnitude
magnitudeUnassigned
count
unassigned
taxon
rank

113254\_out

12

12
superkingdom
2

1239
12
phylum

12
class
91061

186826
12
order

12
family
1300

1301
12
genus


SRS016858\_contig\_number\_11202
1304
species
1

species
11

SRS015274\_contig\_number\_195SRS015985\_contig\_number\_8041SRS017080\_contig\_number\_contig-100\_3684.38258SRS018612\_contig\_number\_195SRS050628\_contig\_number\_contig-100\_15594.15594SRS103943\_contig\_number\_4382SRS104285\_contig\_number\_2947SRS143090\_contig\_number\_contig-100\_411.25051SRS146874\_contig\_number\_1336SRS148096\_contig\_number\_contig-100\_23878.23879SRS148979\_contig\_number\_contig-100\_26038.26039
1343
